# Therapeutic and prophylactic uses of invertebrates in contemporary Spanish ethnoveterinary medicine

**DOI:** 10.1186/s13002-016-0111-1

**Published:** 2016-09-05

**Authors:** José Antonio González, Francisco Amich, Salvador Postigo-Mota, José Ramón Vallejo

**Affiliations:** 1 Grupo de Investigación de Recursos Etnobiológicos del Duero-Douro (GRIRED), Facultad de Biología, Universidad de Salamanca, Salamanca, Spain; 2 Equipo de Antropología Social y Cultural, Facultad de Medicina, Universidad de Extremadura, Badajoz, Spain; 3 Departamento de Terapéutica Médico-Quirúrgica (Facultad de Medicina) – Departamento de Didáctica de las Ciencias Experimentales y de las Matemáticas (Facultad de Educación), Universidad de Extremadura, Badajoz, Spain

**Keywords:** Invertebrates, Ethnozoology, Ethnoveterinary medicine, Zootherapy, Spain

## Abstract

Zootherapeutic practices in ethnoveterinary medicine are important in many socio-cultural environments around the world, particularly in developing countries, and they have recently started to be inventoried and studied in Europe. In light of this, the purpose of this review is to describe the local knowledge and folk remedies based on the use of invertebrates and their derivative products in contemporary Spanish ethnoveterinary medicine. An overview in the fields of ethnozoology, ethnoveterinary medicine and folklore was made. Automated searches in the most important databases were performed. All related works were examined thoroughly and use-reports were obtained from 53 documentary sources. The traditional use of 18 invertebrate species and five ethnotaxa and a total of 86 empirical remedies based on the use of a single species was recorded. The two most relevant zoological groups were found to be insects and molluscs. A broad diversity of body parts or derivative products have been and are used to treat or prevent ca. 50 animal diseases or conditions, in particular diseases of the skin and subcutaneous tissue, different infectious livestock diseases, and disorders of the eye and adnexa. Cattle, sheep and equines form the group of domestic animals in which the greatest number of remedies are mentioned. In addition, seven magical remedies and practices are documented. In comparison with other culturally related areas, this is a rich heritage. The use-reports included here will help in the search for new and low-cost drugs for treating livestock and alternative materials for pharmaceutical purposes, future research addressing the validation of the effects and the development of organic farming.

## Background

Several authors have argued that the medicinal use of animals and products derived from them is a worldwide phenomenon, dating back to prehistoric times and co-evolving with human communities [1, 2]. In this sense, invertebrates and derived products have been used for curing and preventing different diseases affecting humans throughout the world [3–9]. Study of this group of animals, in particular insects, is of great interest owing to the large number of chemical compounds they synthesise [10–12]. According to Cherniack [13, 14], globally ubiquitous invertebrates potentially provide a cheap, plentiful supply of healing substances in an economically challenged world. Likewise, invertebrate-based medicine is coming under increasing scrutiny for its incorporation into evidence-based medicine [12–15].

In view of the above, the use of invertebrates in ethnoveterinary medicine (EVM), the scientific term for traditional animal health care [16, 17], is currently a major topic in an increasing number of research projects (see http://www.ethnovetweb.com/), and the publication of studies on certain territories or reviews in international journals dealing with veterinary medicine or pharmacology is increasingly frequent around the world [18–20].

As in other developed countries, in Spain there are few works addressing local EVM that include animal-based remedies; however, as we have recently highlighted [21], there is valuable information disseminated in numerous anthropological or ethnographical studies.

Thus, the aims of the present review were as follows: (1) to document and analyse local knowledge about the veterinary use of invertebrates and their derivative products in contemporary Spanish EVM; (2) to contribute to the transfer of part of the traditional knowledge to new generations; (3) to contribute to the dissemination of results within the scientific community in order to open a door to research in other disciplines, for example future research into the validation of the effects, and (4) to contribute to the establishment of animal production systems consistent with ecological agriculture and sustainable development.

## Methods

### Data collection

To access the maximum number of documentary sources, a qualitative systematic review of international and national databases was conducted. The ISI Web of Science and Anthropology Plus and JSTOR III—Arts and Sciences international databases were consulted. The national resources referenced include the database of PhD theses, TESEO; the information system of the databases of the CSIC (Spanish Research Council); the Dialnet bibliographic website; Google Scholar, and the catalogue of Public State Libraries. The overall search pattern covered the title, abstract and keywords concerning ethnozoology-related disciplines that have UNESCO codes (e.g. anthropology, the history of veterinary science, zoology) and the terms “invertebrates”, “folk veterinary medicine”, “folklore”, “ethnobiology”, “ethnozoology”, “ethnoentomology”, “ethnoveterinary medicine” and “zootherapy”, in conjunction with the Spanish geographical context. No restrictions regarding the language of the publications consulted were imposed.

### Taxonomy and nomenclature

After performing a thorough analysis of the references retrieved and studied, the data were included in a database with a number of fields to characterise the animal species used, the ailment treated, the geographical location of the remedy and its corresponding bibliographic citation. The vernacular names found were contrasted and subjected to discriminatory analysis following biological, ecological and biogeographical criteria [22, 23].

Regarding animal taxonomy and nomenclature, we followed the Species 2000 & ITIS Catalogue of Life: 2016 Annual Checklist (see www.catalogueoflife.org/annual-checklist/2016/).

In relation to the pathologies of livestock and domestic animals treated, it should be noted that most of the works consulted include popular names to refer to them, names that have been necessary to correspond with the nomenclature used by professionals in animal healthcare. For the proper identification of diseases we have consulted some classic Spanish dictionaries [24, 25] and much of the current veterinary literature [26–32].

## Results and Discussion

### Documentary resources

This review was carried out using data including more than 60 documentary sources from the beginning of the twentieth century to the present. Among them, 53 have allowed the registration of a total of 93 remedies based on the use of a single animal species. As for the type of these 53 sources, we have obtained use-reports from six theses, four of which approach the study of EVM in a particular geographical area, and two belong to the field of ethnobotany. We also obtained data from 26 journal papers, most of them (80 %) published in journals in the field of folklore and ethnography, and 21 books, nine concerning superstition, folklore or ethnobotany, five within the scope of ethnomedicine and only seven directly related to EVM.

In order to assess how contemporary the reviewed veterinary practices are, we mainly obtained use-reports in studies published over the past 15 years, namely from 12 works published between 2001 and 2008 and 13 published between 2010 and 2015. We have also included data collected in 11 works from the 1990s, nine from the 1980s, six from the period 1952–1976, and two from the early 20th century (1907 and 1927).

### General ethnozoological and ethnoveterinary data

**Fig. 1:**
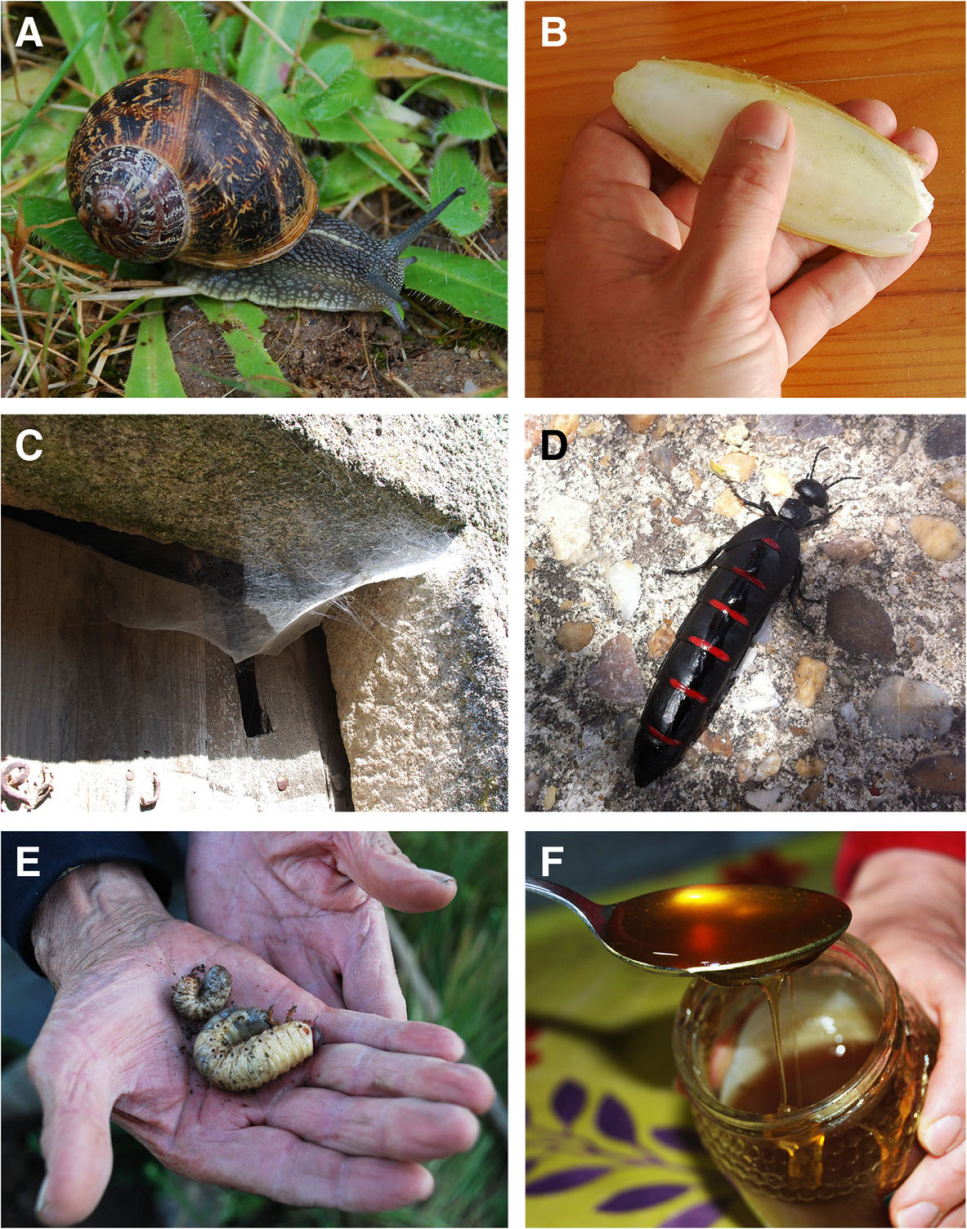
Examples of invertebrates and derivative products used in contemporary Spanish EVM. **a** – brown garden snail (*Cornu aspersum*), **b** – cuttlefish bone, **c** – cobweb, **d** – female of *Berberomeloe majalis*, E – Scarab beetle larvae, F – bee honey (photos by J. A. González)

We recorded the use of 18 species of invertebrates (belonging to 16 zoological families) in Spanish EVM. The terms “snails”, “spiders”, “beetle larvae”, *carcoma* (i.e. “woodworm and powder-post beetles”) and “ants” are considered as “ethnotaxa” (Fig. 1). Table 1 summarises the scientific and vernacular names of the animal species used, and the 93 veterinary remedies documented: 86 of the empirical type and 7 magical.

**Table 1:** Invertebrates used in contemporary Spanish EVM with indications of the body parts and/or products used, ailments treated, target domestic animal groups, modes of preparation and application, and geographical location of the remedies

Animals used (vernacular names)	Part (s) or products used	Diseases or troubles treated (● = current use)	Animal (s) treated	Preparation (administration route)^a^	Remedy type^b^	Geographical location	Ref. No.
MOLLUSCA							
BIVALVIA							
Mytiloida, Mytilidae							
*Mytilus edulis* Linnaeus, 1758 (mejillón)	Shell	Corneal ulcers	All livestock	Pulverised (EX)	EMP-CUR	Comarca de Zafra (Badajoz)	[43]
Ostreoida, Ostreidae							
*Crassostrea gigas* (Thunberg, 1793) (ostión)	Shell	As dietary supplement (**●**)	Poultry	Pulverised (IN)	EMP-PRE	Doñana (Andalusia)	[101]
GASTROPODA							
Stylommatophora [several families]							
Snails (caracoles, cargols, caragols, cascoxos)	Whole animal	Scald, hoof rot	Cows	Fresh (EX)	EMP-CUR	Asturias, Basque Country	[48, 55]
		Splenic fever (anthrax)	Cattle	Fresh (EX)	EMP-CUR	Zumaia (Guipúzcoa)	[48]
	Shell	——— (prophylaxis)	All livestock	Without prepraration (EX)	MAG-PRE	Western part of Asturias	[99]
		Corneal ulcers	All livestock	Burned and pulverised (EX)	EMP- CUR	Comarca de Serrablo (Huesca)	[75]
Stylommatophora, Helicidae							
*Cornu aspersum* (O.F. Müller, 1774) [= *Helix aspersa* O.F. Müller, 1774] (caracol común, caracol de jardín, cargol bover, caragol bover)	Whole animal	Scald, hoof rot	Cows	Fresh (EX)	EMP-CUR	Comarca de Campoo (Cantabria)	[66]
		Keratoconjunctivitis	Sheep	Toasted and crushed (EX)	EMP-CUR	Comarca del Pallars (Lérida)	[69]
		Viper bites	Sheep	Fresh (EX)	EMP-CUR	Comarca del Pallars (Lérida)	[69]
Stylommatophora, Arionidae							
*Arion ater* (Linnaeus, 1758) (babosa negra, limaco, lumaco, llimiagu)	Whole animal	Scald, hoof rot	Cows	Crushed (EX)	EMP-CUR	Bandujo –Proaza– and La Matosa –Piloña– (Asturias)	[55]
	Slime	Choked on food	Cows	Fresh (IN)	EMP-CUR	Ibias (Asturias)	[85, 88–90]
	Entrails	Eye disorders	Cows, sheep, goats, horses	Dried (EX)	EMP-CUR	Ibias (Asturias)	[80]
*Arion hortensis* Férussac, 1819 (babosa, lumiago)	Whole animal	Scald, hoof rot	Cows	Without prepraration (EX)	EMP-CUR	Comarca de Campoo (Cantabria)	[66]
CEPHALOPODA							
Myopsida, Loliginidae							
*Loligo vulgaris* Lamarck, 1798 (calamar)	Internal shell	Corneal ulcers	Sheep, goats	Dried and pulverised (EX)	EMP-CUR	Las Cruces de Gáldar and Cueva Corcho (Gran Canaria)	[76]
		Wounds	All livestock	Dried and pulverised (EX)	EMP-CUR	Santa Olalla de Cala (Sierra de Aracena, Huelva)	[57]
Sepiida, Sepiidae							
*Sepia officinalis* Linnaeus, 1758 (sepia, sípia, jibia, xiba, choco)	Cuttlebone	Corneal ulcers	Cattle, equines, sheep, goats	Dried and pulverised (EX)	EMP-CUR	A Pastoriza (Lugo), Laza (Orense), Gozón and El Franco (Asturias), Comarca de Campoo (Cantabria), Valle de Carranza (Vizcaya), Viniegra de Abajo (La Rioja), Valls d’Aguilar (Lérida), Arànser (Lérida), Puigcerdà (Gerona), Camp de Tarragona	[38, 49, 55, 56, 66, 67, 73, 77–79]
		Pinkeye (infectious bovine kerato-conjunctivitis)	Cattle	Dried and pulverised (EX)	EMP-CUR	Valle de Carranza (Vizcaya), Comarca del Pallars (Lérida)	[67, 69]
		Aphthous fever, hoof-and-mouth disease	Cattle	Dried and pulverised (IN)	EMP-CUR	Comarca del Pallars (Lérida)	[69]
		Udder injuries	Goats	Dried and pulverised (EX)	EMP-CUR	Canary Islands	[72]
Octopoda, Octopodidae							
*Octopus vulgaris* Cuvier, 1797 (pulpo, polbo)	Whole animal	Cough	Pigs	Boiled (IN)	EMP-CUR	Cee (La Coruña)	[73]
ANNELIDA							
CLITELLATA							
Arhynchobdellida, Hirudinidae							
*Hirudo medicinalis* Linnaeus, 1758 (sanguijuela, sangrijuela)	Whole animal	Inflammation of the tongue (glossitis)	Equines	Without prepraration (EX)	EMP-CUR	Villarino de los Aires (Salamanca)	[52]
		Indigestion	All livestock	Without prepraration (EX)	EMP-CUR	Malpartida de Plasencia and Toril (Cáceres)	[64]
		Clostridium infections	Cows	Without prepraration (EX)	EMP-CUR	Comarca de la Sierra de Cádiz (Cádiz)	[71]
		Bruises	All livestock	Without prepraration (EX)	EMP-CUR	Apodaca (Álava)	[39]
		Osteoarticular inflammations	Cows	Without prepraration (EX)	EMP-CUR	Guijuelo (Salamanca)	[52]
		Cerebral congestion	All livestock (mainly cows and pigs)	Without prepraration (EX)	EMP-CUR	Basque Country, Comarca de Zafra (Badajoz)	[42, 100]
Rhynchobdellida, Glossiphoniidae							
*Helobdella stagnalis* (Linnaeus, 1758) (sanguijuela colorada, sanguijuela roja)	Whole animal	Osteoarticular inflammations	All livestock	Without prepraration (EX)	EMP-CUR	Doñinos de Salamanca (Salamanca)	[52]
ARTHROPODA							
ARACHNIDA							
Araneae [several families]							
Spiders (arañas)	Cobwebs	Haemorrhage (wounds) (**●**)	All livestock	Without prepraration (EX)	EMP-CUR	Tierra de Cameros (La Rioja), Province of Guipúzcoa, Basque Country, Valls d’Aguilar (Lérida), Comarca de Zafra (Badajoz), Extremadura, Estepona (Málaga), Almendra, Cabeza de Framontanos, La Calzada de Béjar and Garcihernández (Salamanca), Valle de Tena and Tierra de Biescas (Huesca)	[38, 43, 47–54, 94]
		Healing scrotal wounds (after castration)	All livestock	Without prepraration (EX)	EMP-CUR	Basque Country	[51]
		Caudectomy	Sheep	Without prepraration (EX)	EMP-CUR	Taja –Teverga– (Asturias), Valle de Carranza (Vizcaya)	[55, 56]
		Dehorning (by amputation)	Calves	Without prepraration (EX)	EMP-CUR	Basque Country	[47, 48]
		Broken horn	Cattle	Without prepraration (EX)	EMP-CUR	Many localities of the province of Salamanca	[52]
		Broken leg	Sheep	Without prepraration (EX)	EMP-CUR	Santa María de Sando (Salamanca)	[52]
		Scald, hoof rot	Sheep	Without prepraration (EX)	EMP-CUR	Almendra (Salamanca)	[52]
		Corneal ulcers	Cows	Without prepraration (EX)	EMP-CUR	Saelices el Chico (Salamanca)	[52]
Scorpiones, Buthidae							
*Buthus occitanus* (Amoreux, 1789) (escorpión, alacrán)	Whole animal	Colds	Equines	Bolied (EX)	EMP-CUR	Bárcabo (Huesca)	[95]
		Urinary retention	All livestock	Maceration in olive oil (EX)	EMP-CUR	La Aparecida –Orihuela– (Alicante)	[96]
		Anti-cystitic or against ureteral obstruction	Mules	Fried in olive oil (IN)	EMP-CUR	Castile-La Mancha	[98]
		Scorpion stings	All livestock	Maceration in alcohol (EX)	EMP-CUR	Monzón (Huesca)	[95]
			All livestock	Crushed (EX)	EMP-CUR	Monzón (Huesca)	[95]
*Buthus ibericus* Lourenço & Vachon, 2004 (escorpión, alacrán, arraclán, araclán, aracrán)	Whole animal	Aphthous fever, hoof-and-mouth disease	Cattle, goats	Without prepraration (EX)	MAG-PRE	Comarca de El Monfragüe (Cáceres), El Bodón, El Cabaco, Cabeza de Framontanos, Membribe de la Sierraand Sepulcro-Hilario (Salmanca)	[52, 64]
		Disorders of lacrimal system (dacryocystitis, canaliculitis, stenosis)	All livestock	Without prepraration (EX)	MAG-CUR	Alburquerque (Badajoz)	[62]
			Sheep	Without prepraration (EX)	MAG-CUR	Los Ibores and the Valle del Alagón (Cáceres)	[50]
		Equine colic	Equines	Fried in olive oil (EX)	EMP-CUR	Gallegos de Argañán (Salamanca)	[52]
		Urinary retention	Foals	Maceration in olive oil (EX)	EMP-CUR	Comarca de Zafra (Badajoz)	[43]
			All livestock	Fried in olive oil (IN)	EMP-CUR	Montes de Toledo (Toledo)	[97]
		Wounds	All livestock	Fried in olive oil (EX)	EMP-CUR	Almendra (Salamanca)	[58]
			All livestock	Maceration in olive oil (EX)	EMP-CUR	Aldeatejada (Salamanca)	[52]
			Cows	Fried in olive oil (EX)	EMP-CUR	Morasverdes (Salamanca)	[52]
		Antiseptic after castration	Pigs	Fried in olive oil (EX)	EMP-CUR	Almendra and Berrocal de Huebra (Salamanca)	[52, 59, 60]
		Scorpion stings	Oxen	Fried in olive oil (EX)	EMP-CUR	Las Veguillas and Peña de Cabra (Salamanca)	[99]
			Sheep	Crushed and fried in olive oil (EX)	EMP-CUR	Extremadura	[50]
			Cattle	Without prepraration (EX)	MAG-PRE	Encinas de Arriba (Salamanca)	[52]
INSECTA							
Coleoptera, Lucanidae							
*Lucanus cervus* (Linnaeus, 1758) (vacaloura, vacallorina)	Mandibles of the male (and head)	Viper bites	All livestock	Dried (EX)	MAG-PRE	Asturias	[85]
Coleoptera, Meloidae							
*Meloe proscarabaeus* Linnaeus, 1758 (carraleja)	Haemolymph	Inflammation (by trauma)	Cows	Fresh (EX)	EMP-CUR	Cantabria	[91]
*Berberomeloe majalis* (Linnaeus, 1758) (aceitero/a, papaceite, curica, curita, curato, curacurato, curacurate, curacuracho, curapacho, fraile, médico, tabernero/a, vaquita, avaón, quilisón, vinatero, vinagrera)	Haemolymph	Warts	All livestock	Fresh (EX)	EMP-CUR	Comarca de Zafra (Badajoz)	[43]
		Genital warts (in males)	Equines	Fresh (EX)	EMP-CUR	Comarca de Zafra (Badajoz), Malpartida de Plasencia (Cáceres)	[43, 64]
		Lumps in articulations	Equines	Fresh (EX)	EMP-CUR	Madroñera (Cáceres)	[92]
			All livestock	Fresh (EX)	EMP-CUR	Malpartida de Plasencia, Serrejón, Jaraicejo, Torrejón el Rubio and Serradilla (Cáceres)	[64]
			All livestock	Fresh (EX)	EMP-CUR	Comarca de Zafra (Badajoz)	[43]
		To interrupt breastfeeding (weaning)	Calves	Fresh (EX)	EMP-PRE	Benalup de Sidonia (Cádiz)	[63]
		To heal infected wounds (particularly in the legs)	All livestock	Fresh (EX)	EMP-CUR	Garrovillas, Malpartida de Plasencia, Navas del Madroño, Serrejón, Jaraicejo, Torrejón el Rubio and Serradilla (Cáceres), La Muela (Cádiz), Sanlúcar del Guadiana (Huelva)	[63, 64]
	Whole animal	Wounds (**●**)	All livestock	Maceration in olive oil (EX)	EMP-CUR	Western part of the province of Granada, Berganciano (Salamanca)	[52, 61, 65]
		Chafing	Equines, cows, oxen	Fried in olive oil (EX)	EMP-CUR	Aldea del Obispo (Salamanca)	[52]
		Viper bites	Sheep, goats	Without prepraration (EX)	EMP-CUR	Viniegra de Abajo (La Rioja)	[38]
Coleoptera, Scarabaeidae							
Scarab beetles larvae (gallinas ciegas, gallinetas, cocos, roscas, chicharras, gusanos del estiércol, gusanos de las basuras, gusanos del pasmo)	Whole animal (larva)	Wounds and chafing	Equines, cows, oxen	Fried in olive oil or roasted (EX)	EMP-CUR	Aldea del Obispo, Fuenteguinaldo, Membribe de la Sierra and Mieza (Salamanca)	[52]
		Antiseptic after castration	Pigs	Fried or macerated in olive oil (EX)	EMP-CUR	Alburquerque and Comarca de Zafra (Badajoz), Western part of the province of Granada, Almendra, El Bodón, Saelices el Chico, Villar de Ciervo and Villarino de los Aires (Salamanca)	[43, 52, 61, 62]
Coleoptera [several families]							
Woodworm and powderpost beetles (carcoma)	Frass (a mixture of sawdust and excrement)	Scald, hoof rot	Cows	Kneaded together with olive oil (EX)	EMP-CUR	Valle de Carranza (Vizcaya)	[67]
		Chafing	Equines	Without prepraration (EX)	EMP-CUR	Basque Country	[51]
Hymenoptera, Formicidae							
Ants (hormigas)	Whole animal	Clostridium infections	All livestock	Without prepraration (IN)	EMP-CUR	Province of Badajoz (near Sierra de Aracena)	[77]
		Meteorism	Cows	Without prepraration (EX)	EMP-CUR	Bustiellu –Proaza– (Asturias)	[55]
		Indigestion	All livestock (mainly cows)	Bolied (IN)	EMP-CUR	Asturias, Betanzos (La Coruña)	[73, 85]
		To promote pregnancy	Cows	Bolied (IN)	EMP-CUR	Curtis (La Coruña)	[73]
		Mastitis	Cows	Without prepraration (EX)	MAG-CUR	Betanzos and Cesuras (La Coruña)	[73]
*Formica rufa* Linnaeus, 1761 (hormiga roja)	Whole animal	To facilitate rumination	Cows	Without prepraration (IN)	EMP-CUR	Comarca de Campoo (Cantabria)	[66]
Hymenoptera, Vespidae							
*Vespa crabro* Linnaeus, 1758 (avispa, tártago)	Whole animal	Indigestion	All livestock	Without prepraration (IN)	EMP-CUR	Asturias	[85]
Hymenoptera, Apidae							
*Apis mellifera* Linnaeus, 1758 (abeja, abeja melífera)	Honey	Pasteurellosis	Cows	Raw (IN)	EMP-CUR	Berrocal de Huebra (Salamanca)	[60]
		Aphthous fever, hoof-and-mouth disease (**●**)	All livestock	Raw and mixed with vinegar (EX)	EMP-CUR	Valle de Tena and Tierra de Biescas (Huesca)	[54]
		Scald, hoof rot	Sheep, goats	Raw (EX)	EMP-CUR	Tierra de Cameros (La Rioja)	[38]
			All livestock	Raw and mixed with vinegar (EX)	EMP-CUR	Nocito (Huesca)	[68]
		Intestinal parasites	Equines, cattle	Raw and mixed with ginger and sodium carbonate (IN)	EMP-CUR	Sarria (Lugo)	[73]
		Pneumonia	Cows	Raw and mixed with pig fat and water (IN)	EMP-CUR	Ameixenda and Cee (La Coruña)	[73]
			Canaries	Raw (IN)	EMP-CUR	Sarria (Lugo)	[73]
		Colds	Cows	Raw and mixed with water (IN)	EMP-CUR	A Pastoriza (Lugo)	[79]
		Corneal ulcers	All livestock	Raw (EX)	EMP-CUR	Asturias	[55]
			Cows	Raw (EX)	EMP-CUR	Comarca de Campoo (Cantabria)	[66]
		Ocular contusions	All livestock	Raw and mixed with water (EX)	EMP-CUR	Comarca de Serrablo (Huesca), Fonsagrada (Lugo)	[73, 75]
		Digestive disorders	Cows	Raw and mixed with bran (IN)	EMP-CUR	Province of La Coruña	[73]
		Stomach cramp	All livestock	Raw (IN)	EMP-CUR	Province of Salamanca	[87]
		Abscess	Sheep, goats	Raw (EX)	EMP-CUR	Tierra de Cameros (La Rioja)	[38]
		Mastitis	Cows, ewes	Raw (EX)	EMP-CUR	Valle de Carranza (Vizcaya), Comarca de La Campiña (Guadalajara), province of La Coruña	[67, 73, 93]
		Wounds and cracks in the udders	Ewes	Raw (EX)	EMP-CUR	Extremadura	[50]
	Beeswax	Constipation	Lambs, kids	Manufactured –candels– (IN)	EMP-CUR	Enclave de Treviño (Burgos)	[86]
		Cracks in the udders	Cows	Without prepraration (EX)	EMP-CUR	Comarca de Campoo (Cantabria)	[66]

^a^ Administration route: EX = external use; IN = internal use

^b^ Remedy type: EMP = empirical; MAG = magical / PRE = preventive healthcare; CUR = curative care

The value of this useful species richness is much greater than that of the single species of invertebrates collected for our country by Souto *et al*. in their world overview [19].

The two most relevant zoological groups in terms of their contribution to EVM in Spain are insects (41 remedies, 6 useful species and 3 ethnotaxa) and molluscs (19 remedies, 8 useful species and 1 ethnotaxon).

Forty-four remedies (47 %) are based on the use of the whole animal. However, many animal products are used as therapeutic resources in Spanish EVM: shells, slime, haemolymph, etc. (see Table 1). Bee honey and cobwebs are the most commonly employed derivative products, being used in a total of 24 remedies (26 %).

As in human medicine [12, 13, 15], honey is a highly reputed product in current EVM, especially in the treatment of wounds and eye infections [33, 34].

In Spanish ethnomedicine, cobwebs have formed part of the arsenal of traditional remedies since time immemorial [35], but in Spain they are also considered to be useful for domestic animals. According to popular belief they protect animals against all diseases and this is why they are never removed from stables [36–40]. There is a proverb that says: *Cuadra sin arañas, bestias nunca sanas* (lit. “A stable without spiders holds no healthy animals”) [41] and, for example, around the first third of the last century in the Merindad de Tudela area (Navarra), the cobwebs were never removed from stables owing to the belief that they prevented stomach cramps in sheep and equines [42].

The majority of remedies (87) are of the curative type, and they are applied externally in 69 cases (79 %) and internally in 18 (21 %). Zootherapeutics are usually applied in simple ways, mostly through direct application to the affected area and without preparation. Note should be taken, too, of the use of olive oil in the preparation of 16 remedies. Olive oil is a very important therapeutic resource in all cultures, both in ethnomedicine and EVM [43–46]. As well as being an extraction medium for active ingredients from animals, the chemical composition of this oil is rich in flavonoids, secoiridoids, iridoids, flavanones, biophenols, triterpenes, benzoic acid derivatives and isochromans, and these offer added medicinal value since they account for its anti-inflammatory, immunomodulatory, analgesic, antimicrobial, antinociceptive, and wound-healing activities among others [46].

In addition, the animal resources documented are used to treat or prevent ca. 50 animal diseases or complications. More than half of the species, and ethnotaxa, are reported for the treatment of more than one ailment. Several species (6) are used for multiple veterinary purposes (at least four). For instance, *Hirudo medicinalis* and *Berberomeloe majalis*, together with products derived from *Apis mellifera* (honey and beeswax), are the most versatile zootherapeutic resources (see Table 1).

### Traditional veterinary medicinal practices

Thirty-one of the 93 documented remedies (33 %) are used to treat all types of livestock in general, not for any single domestic animal group in particular.

**Fig. 2:**
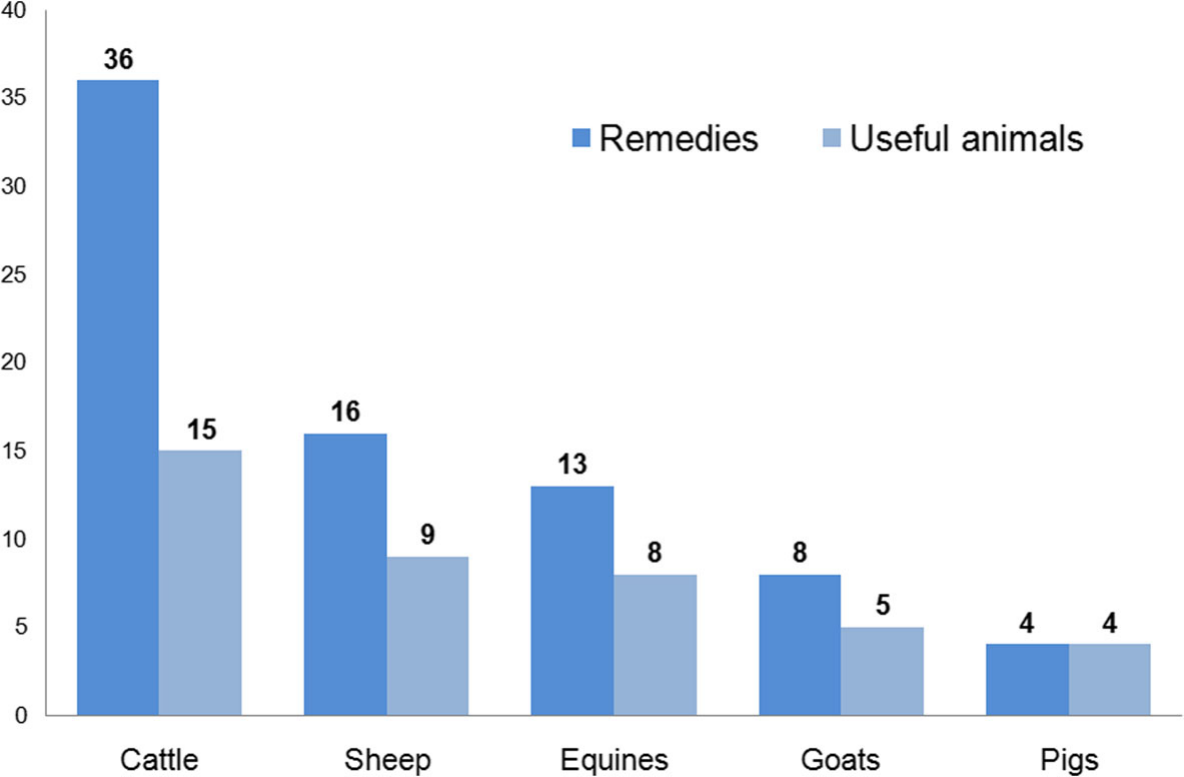
Number of remedies documented and invertebrates used in the care and treatment of specific domestic animal groups

In accordance with their economic importance in Spain, cattle, sheep and equines are the groups of domestic animals for which the greatest number of remedies are mentioned (35, 16 and 13 respectively). The use of invertebrates to treat goats and pigs is also documented (Fig. 2), as well as a curative remedy for canaries.

**Fig. 3:**
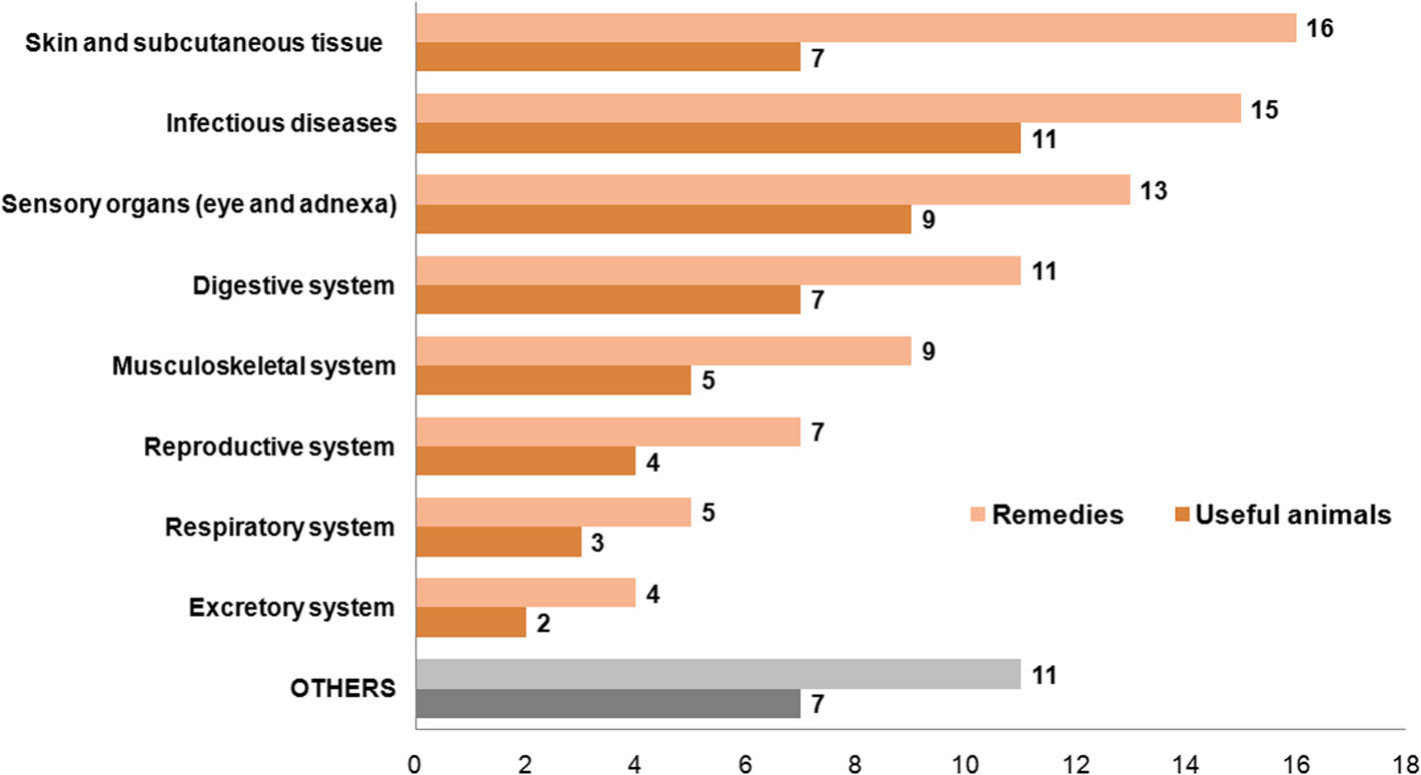
Number of remedies documented and invertebrates used per disease category

The remedies refer to eight main categories of therapeutic use (Fig. 3). The most frequent indications are diseases of the skin and subcutaneous tissue (16 remedies, 7 useful animals), different infectious livestock diseases (15 remedies, 11 animals), and disorders of the eye and adnexa (13 remedies, 9 animals).

#### Skin and subcutaneous tissue (injuries)

Cobwebs are undoubtedly the most important product of animal origin in Spanish EVM for staunching haemorrhages caused by wounds or amputations. Their use as a haemostatic agent extends throughout the country [38, 43, 47–54]. After the wound has been washed with water, cobwebs are applied directly to the wound, as a dressing. It is also claimed that that are a good anti-scarring agent; they form a cap similar to fibrin on the surfaces to which they are applied. In the Basque Country, cobwebs used to be placed on the cuts made to the scrotum after castration as a healing and antiseptic agent [51] and for healing dehorning (by amputation) in calves [47, 48]. After the wound had been washed with water, the cobwebs were placed over it and the abrasion was bandaged. On successive days the affected area was subjected to cures with hydrogen peroxide and cobwebs again [47]. Similarly, when they were cutting off the tails of lambs the shepherds from Teverga (Asturias) and the Valle de Carranza (Vizcaya) used to place cobwebs on the site of the cut [55, 56].

We have also documented other remedies for treating the wounds of domestic animals. Drying and grinding the internal shell of the common squid (*Loligo vulgaris*) and applying the powder to wounds was a common practice in the Sierra de Aracena (Huelva) [57]. In the province of Salamanca we have documented three remedies based on the use of scorpions; in Almendra, farmers would apply the oil used to fry the leaves of wall pennywort – *Umbilicus rupestris* (Salib.) Dandy, Crassulaceae – together with a whole scorpion to make an ointment that they applied with a stork feather [58]; in Morasverdes, they would place a squashed scorpion on the wounds of cattle brought in due to fighting (with their horns) and at Aldeatejada they used to put several scorpions in olive oil to macerate, and with this oil, which was kept in the house, they would treat the animals’ wounds [52]. Also in the province of Salamanca, at the beginning of the twentieth century a common practice was to fry several scorpions (usually three), which were then spread on the wound a couple of times a day [52, 59, 60]. For the same purpose, in the provinces of Badajoz, Granada and Salamanca the farmers would fry or macerate several fat larvae from beetles of the family Scarabaeidae in olive oil, which was then used as an anti-inflammatory and healing agent, applied before and after castration [43, 52, 61, 62]. The same larvae were used in several villages in the province of Salamanca to treat wounds and chafing on draught animals, mainly on their legs. To do this, farmers would oil-fry or roast several larvae in a pan and spread the fat they exuded on the animals’ wounds [52].

In the south-western quadrant of Spain, to heal infected wounds (particularly on the legs) the recommendation was to cut off the head of a blister beetle, *Berberomeloe majalis*, and spread the haemolymph on the wound [63, 64]. By contrast, in the western part of the province of Granada and at Berganciano (Salamanca) a medicinal preparation was made by macerating many specimens of this beetle in olive oil [52, 61, 65]. At Aldea del Obispo (Salamanca) farmers used to collect several specimens and fry them in olive oil and use this to cure the chafing due to the tack of draught animals [52].

In the Basque Country the dust emerging from woodworm burrows (i.e. frass, a mixture of sawdust and excrement) due to the exit of the imagos of woodworm and powder-post beetles from their burrows was applied directly to chafing caused in equines by tack [51].

Finally, in Tierra de Cameros (La Rioja) shepherds would treat the abscesses on sheep and goats by spreading these abundantly with honey [38].

#### Infectious diseases

At Berrocal de Huebra (Salamanca) in order to cure cows affected by pasteurelosis, livestock raisers used to put a stick dipped in honey in the animal’s mouth (tying it there with string around the horns). The effect sought was that the animal would move its tongue, savouring the honey, hence increasing the animal’s salivation [60].

In Asturias, Cantabria and the Basque Country scald or hoof rot, the ruminant hoof disease caused by *Fusobacterium necrophorum*, is treated by applying a snail-based poultice on cows. Farmers would tie a piece of cloth full of snails onto the animal’s hoof and would leave it there for a few days [48, 55, 66]. In these same Autonomous Communities, slugs were used to cure infections; thus at Proaza and Piloña (Asturias) farmers would apply poultices with verdigris, salt and squashed black slugs (*Arion ater*) [55], and in the District of Campoo (Cantabria) they would tie a bag of slugs (*A. hortensis* in this case) on the leg of the animal and leave it there for a few days [66]. By contrast, in the Valle de Carranza (Vizcaya) just a few decades ago farmers would mix olive oil and the frass of woodworm and powder-post beetles. They would then knead this until they obtained a whitish paste with the consistency of an ointment and apply it to wounds [67]. The shepherds in Tierra de Cameros (La Rioja) have traditionally applied abundant honey to the hooves of their sheep and goats [38]; shepherds in Nocito (Huesca) rubbed, once or twice on alternate days, the legs of their animals with an ointment prepared with 250 g of honey, red wine vinegar and five tablespoons of copper sulphate [68], and when their sheep’s hooves were very long and deformed the shepherds at Almendra (Salamanca) would cut them off and treat them with cobwebs [52].

In the region of Pallars (Lérida) aphthous fever was cured with powders for internal use with the following ingredients: a cuttlebone (the internal shell of *Sepia officinalis*), incense, wheat bran, a piece of the snake *Hierophis viridiflavus* (Lacépède, 1789) and toasted eggshells. These ingredients were ground and mixed with salt, the major component of the medicine, and were given to cattle in a single dose [69]. For their part, farmers in the mountains of Huesca prepare a kind of hyssop with honey and vinegar, putting this mixture around a stick and allowing it to cool. Then they passed the hyssop mixture across the mouth of the sick animal, for disinfection [54]. The preventive remedy used in the provinces of Cáceres and Salamanca was very different, where scorpions (always an odd number, as a ritualistic component) were placed in a cowbell without a clapper and sealed with cork. The bell thus prepared was hung round a sick animal’s neck. When the scorpions died and dried up, the group of animals was believed to be protected [52, 64]. In the Monfragüe district, farmers claimed that sick goats could be cured by putting 15 scorpions in a cowbell around their necks [64].

Known also as anthrax, splenic fever is a serious bacterial infection that mainly affects cattle. To cure this disease, in Zumaia (Guipúzcoa) a poultice of crushed terrestrial snails, mixed with resin, a few drops of wine, a piece of church candle, and a bit of olive oil were used. All this was heated and passed through a cloth. Once cool the resulting ointment was applied to the sick animal [48].

In Badajoz, at the beginning of the twentieth century, infections caused by *Clostridium* were cured by giving powdered ants to the animals to make them break wind [70]. In the Sierra de Cádiz, bloodletting with medicinal leeches (*Hirudo medicinalis*) was performed to treat infected cows [71].

Finally, in Extremadura, warts on animals were treated by rubbing a specimen of *Berberomeloe majalis* directly or mixed with oil on the affected part [43]. In the case of genital warts in male equines the beetle’s haemolymph was used to treat the affected area [43, 64].

#### Sensory organs (eye and adnexa)

To alleviate pain in cases of ocular contusions, in Huesca and Lugo honey was spread on the eye of the animal [72, 73].

To cure corneal ulcers caused by eye lesions, mainly scratches and cuts from branches or vegetation in general, in Asturias and Cantabria the eyes were also treated with honey [55, 66], and at Saelices el Chico (Salamanca) a cobweb was placed liberally on the affected eye [52]. However, such ocular lesions have usually been treated in Spain by administering (on the eye) a fine powder made of different animal elements. At Zafra (Badajoz) the valves of the shell of the common mussel (*Mytilus edulis*) were ground to a fine powder and sprinkled directly on the affected eye [43]. It is highly likely that in the interior areas of the country traditional use involved river mussel shells, of species such as the painter’s mussel, *Unio pictorum* (Linnaeus, 1758), or the swan mussel, *Anodonta cygnea* (Linnaeus, 1758), which can still be found in the south-east of the province of Badajoz [74]. In the District of Serrablo (Huesca) snail shells were gathered and roasted on coals. At first they turned black, and then white, after which they were ground and sieved, until a very fine powder was obtained. A tube of rolled-up paper was then filled with the powder, which was blown into the eye of the animal. It was claimed that this cure should be applied for nine consecutive days [75]. On the island of Gran Canaria, shepherds treated their sheep and goats with *mierda de gaviota*, i.e. the internal shell of *Loligo vulgaris*. They would scrape this with a knife and place the powder in the eye of the sick animal [76]. Despite this, the most common remedy was to put powder obtained by scraping a cuttlefish bone into the eye of sick animals. The powder was placed on a piece of paper, which was rolled up, and the powder was blown directly into the bad eye. In many districts and regions in the north of Spain this simple remedy was practised daily until the animal was cured [38, 49, 55, 56, 66, 67, 73, 77–79].

Likewise, to combat infectious bovine keratoconjunctivitis, also known as “pinkeye”, caused by the bacterium *Moraxella bovis*, in the Valle de Carranza (Vizcaya) and the Pallars region (Lérida), shepherds would blow the powder obtained from finely ground cuttlebone into the bad eye through a thin paper tube [67, 69].

In this same Catalan region, to treat keratoconjunctivitis in sheep, four or five *caragols bovers* (*Cornu aspersum*) were roasted in coals together with a stem of cabbage. After crushing all together, a pinch of salt was added. The mixture was sieved and applied to the conjunctiva of the sick animal [69].

In order to treat disorders of the lacrimal system (dacryocystitis, canaliculitis, stenosis) in Alburquerque (Badajoz) a scorpion was placed in a bell, sealed with cork, and hung from the neck of the sick animal [62]. Shepherds in the north of the province of Cáceres used to hang a needle case containing a scorpion around the sick animal’s neck [50]. In both cases, the scorpion and the eye problem were assumed to “dry out” at the same time.

Towards the middle of the last century, in Ibias (Asturias), to treat any type of ocular disorder, the animal’s eye was opened and home-made medicine was applied; this was a mixture of different plant products (rose petals, rosemary, garlic, rue, etc.) together with the innards of a black field slug [80]. In this case, the remedy has strong associated symbolisms. Plants considered to be universal panaceas are used such as rosemary, which appears in classic works such as Cervantes’ *Don Quixote* [81] and in pharmacopoeias such as the Hispana from 1794 (*Spiritus Rosmarini* or *Aqua Reginae Hungariae*), together with other plants with an apotropaic effect used to treat psychological problems, such as rue or rose petals [82, 83], all combined with the innards of a black mollusc. Disease is black and dirty, as opposed to the whiteness of health, and this is why antidotes tended to be dark in colour [84].

#### Digestive system

At Villarino de los Aires (Salamanca) when an equine suffered from glossitis a medicinal leech was placed with great care on the animal’s tongue to reduce the inflammation [52].

In Proaza (Asturias), to combat meteorism, an ant colony (with earth included) was placed in a bag and then placed on the sick cow in the area of the spleen. This was a reputable remedy [55]. By contrast, in the Campoo (Cantabria), cows were given red wood ants (*Formica rufa*) to eat when their digestive tract was blocked, to facilitate rumination [66].

A remedy recommended to cure indigestion in Asturias and Galicia was to put an ants’ nest in water and boil it, giving the resulting drink to the sick animal [73, 85]. In Asturias it was also claimed that a complete cure could be obtained with a purge of hornets (*Vespa crabro*) [85]. The inhabitants of Malpartida de Plasencia and Toril (Cáceres) recommended that the sick animal should be bathed over several consecutive days in a pool with an abundance of leeches [64].

In Treviño (Burgos) lambs and kids with constipation were treated by inserting beeswax candles into their rectums [86], and in Salamanca sick animals with stomach cramps were made to eat a specified amount of honey [87].

At Ibias (Asturias), when a cow choked (on a large piece of apple for example) and the ministrations of the local *curandero* (healer) had no effect, in order to force the foreign object out of the mouth or pass down the gullet of the animal the inhabitants would prepare a lubricant compound with different plant components (cloves of garlic, rosemary, parsley, etc.), white wine, oil, milk, holy water and the slime from a black slug (as a ritualised element). The resulting liquid, once sieved, was placed in a bottle for giving to the sick animal [85, 88–90].

At Gallegos de Argañán (Salamanca) the inhabitants would fry scorpions and use the oil to rub the bellies of equines suffering from equine colic [52].

In the province of La Coruña beef farmers cured any kind of digestive disorder by forcing cows to take honey mixed with bran [73].

#### Musculoskeletal system

The haemolymph of *Meloe proscarabaeus* was applied by cattle ranchers in Cantabria on inflamed areas (due to trauma), as a resolutive [91], whereas in Extremadura the haemolymph of *Berberomeloe majalis* was used to cure lumps in articulations, either by rubbing the beetle directly on the affected part or applying the medicinal oil prepared with many specimens macerated in olive oil [43, 64, 92].

At Apodaca (Álava) bloodlettings were performed with medicinal leeches to treat bruises on livestock [39], and in the province of Salamanca common medicinal leeches or *sanguijuelas rojas* (duck leeches, *Helobdella stagnalis*) would be used on the legs of animals (mainly cows) affected by osteoarticular inflammation [52]. In both cases, this was believed to “draw out the bad blood”.

The shepherds of Santa María de Sando (Salamanca) would cover the broken leg of a sheep with large, “dirty” cobwebs from stables. They would then tie splints around the broken limb with a cloth bandage [52]. And in many localities of this province when a cow broke a horn they would cover the stump with abundant cobwebs from stables (“large and dirty, so that the wound will not become infected”) [52].

#### Reproductive system

In the province of La Coruña there was a strange relationship between the health of the cows and ants. Cows are forced to eat ants boiled in water to promote their pregnancy, and a curious ritual was used to treat mastitis: fresh milk was taken from the sick cow and poured over an anthill (preferably over one located on the road towards a church). After doing this, the container in which the milk was transported was broken in the same place and the fragments were left there [73].

Also in La Coruña, as well as in the Valle de Carranza (Vizcaya) and the District of La Campiña (Guadalajara), mastitis in cows and ewes was treated by washing the udders well with cold water and then applying honey [67, 73, 93].

Similarly, in Extremadura, to clean and cure wounds and cracks in the udders of ewes, shepherds would rub honey on them [50], while in the District of Campoo (Cantabria) a salve prepared with beeswax and rosemary was spread on the udders [66], and in the Canary Islands the powder obtained by grinding a cuttlebone was directly applied to injuries on goats’ udders [72, 94].

To interrupt breastfeeding in calves at Benalup de Sidonia (Cádiz) the shepherds would cover the udders of cows with the haemolymph of *Berberomeloe majalis* [63].

#### Respiratory system

Against colds, in Bárcabo (Huesca) scorpions were captured live and boiled, after which the liquid was applied to the chests of sick equines [95]. In A Pastoriza (Lugo) water with honey was given to cows to drink [79].

To combat cough, in Cee (La Coruña) water in which a common octopus had been boiled (*Octopus vulgaris*) was given to pigs to drink [73].

Also in this province, to cure pneumonia sick cows are forced to take honey mixed with pig fat and water [73]. Curiously, in Sarria (Lugo) honey was also used internally as an effective remedy to combat pneumonia in canaries [73].

#### Excretory system

We have documented two remedies against urinary retention based on the use of scorpions: one or several of these arachnids (always an odd number) would be placed in a bottle containing olive oil. They were allowed to macerate and the oil was then used to rub on the belly of the sick animal, or the penis in the case of colts [43, 96]. Alternatively, eggshells would be fried with onion, laurel leaves and a scorpion; a loose paste was made of this and given to the sick animal to drink [97]. In both cases, it was believed that the affected animal would be able to urinate again after a few hours. Also basing themselves on the use of scorpions, as an anticystitic or to treat ureteral obstruction in Castile-La Mancha shepherds would use the oil obtained from frying two or three whole scorpions in olive with laurel leaves. The filtered oil was given to mules to drink [98].

#### Others

At Monzón (Huesca) scorpion stings were treated by applying the liquid resulting from the maceration of a scorpion in a bottle containing alcohol to the area of the sting or by applying the crushed body of the scorpion responsible for the sting directly to the area of the sting [95]. At Las Veguillas and Peña de Cabra (Salamanca) the scorpion that had stung an animal was caught and fried in oil, and the resulting salve was applied to the area of the sting [99]. Similarly, in Extremadura, shepherds would crush and fry the perpetrating scorpion and put the oil on the sting area [50]. To prevent stings, the cattle ranchers of Encinas de Arriba (Salamanca) would place a scorpion inside a cowbell and seal it with a piece of cork. This ritual was believed to protect the cow [52].

In the case of viper bites, at Viniegra de Abajo (La Rioja) shepherds used to place a specimen of *Berberomeloe majalis* on the bite areas of their sheep and goats. This would cure the animal once the area had been punctured and the venom drawn out [38]. In the Pallars region the shepherds, after removing the venom through a cut, applied a poultice made with several snails (with shell), three cloves of garlic, salt and olive oil to the bitten sheep [69]. In Asturias, what is known as *cuerno de la vacaloura*, i.e. the mandibles (and head) of the male stag beetle (*Lucanus cervus*), was a traditionally highly valued amulet for the prevention of snake bites [85].

To eliminate intestinal parasites in Sarria (Lugo) equines and cattle were given a mixture of honey, ginger and sodium carbonate to take [73].

In the Basque Country, in the case of *golpe de sangre* (“blood hit”, cerebral congestion) or paralysis, pigs were bled by placing medicinal leeches on their ears, and on the dewlaps of cattle [100]. At Zafra (Badajoz) leeches were placed on the neck of any animal with cerebral congestion [43].

In the western part of Asturias, farmers used to hang the shell of a snail around the neck of the animals to protect them against diseases in general [99].

Finally, in Doñana (Andalusia) the inhabitants would grind a large calcareous shell of the oyster *Crassostrea gigas* for use on poultry farms as a supplement to poultry feed [101].

### Current uses

Sixty of 93 documented remedies (~66 %) were collected in documentary sources published in this century. Only 31 remedies (33 %) were gathered from those published over the last six years (2010–2015). Among those works, in a very few cases the authors used the present verbal form, just to refer to the veterinary use of honey or cobwebs [51, 54]. Another currently valid practice is the external application of the medicinal oil prepared with many *Berberomeloe majalis* specimens macerated in olive oil [65]. No magical remedies or rituals are still practised. Most veterinary uses and practices documented in this review only remain alive in the memory of elderly people.

### Potential veterinary uses

As we have already mentioned above, bee honey is a highly reputed derivative product in current EVM. The use of honey for preparing ethnoveterinary herbal medicines is widespread but it is used as a single element too. For example, it is used in the treatment of wounds [34] and eye problems [33]. Likewise, at present there are numerous scientific evidences supporting its use in human medicine [102–104]. Therefore, we believe that it is very necessary to develop scientific projects aimed at validating traditional veterinary remedies based on the use of honey and developing its use in clinical practice.

The protein structure of spider silk and its exceptional mechanical properties are the main subject of many investigations regarding its potential biomedical applications. This is a very promising biomaterial in fields such as tissue engineering, because, unlike traditional inert implants, it stimulates growth and the natural activity of the cells in contact with it [105–107]. Spider silk is resistant, biocompatible and biodegradable [106]. Fernández-d’Arlas [108] recently proposed that progress in the understanding of the effects of ions on spider silk could expand its use by developing ointments, vesicles for controlled release systems or cellular substrates. Its role in wound healing constitutes a research field that should certainly be developed.

Like other blister beetle species, *Berberomeloe majalis* secretes cantharidin, a potent vesicant terpene (blistering agent) which has a long history in ethnomedicine. For example, in dermatology, topical cantharidin has long been used to treat warts and molluscum contagiosum [109]. In the field of modern veterinary medicine, cantharidin has been demonstrated to act as a vasoconstrictor and positive inotrope [109].

Moreover, the importance of some products derived from terrestrial snails in different aspects of human and veterinary medicine [14, 110] should be pointed out at this point. In relation to molluscs, although today it is not used in Spanish EVM, another interesting product is cuttlebone. The internal shell of cuttlefish is composed primarily of aragonite (crystal forms of calcium carbonate) and it has a very elaborate architecture [111]. Commonly used as a calcium-rich dietary supplement for caged animals (birds, turtles, snails), it is also an important natural material in Biotechnology [111]. From time immemorial, cuttlebones have been ground up to make polishing powder, which was used for medicinal purposes as an antacid and as an absorbent for treating sore skin. Recently, antibacterial activity [112], bone healing properties [113] and wound healing activity on skin ulcer lesions [114] of cuttlebone have been shown. In addition, Lee *et al*. [115] found that cuttlebone extract induces acute inflammation and promotes cell proliferation.

### A cross-cultural comparison

Remedies based on animals have an ancestral origin, and throughout history have been gathered in the main reference works on pharmacology. With respect to the issues addressed here, invertebrates formed an important part of the therapeutic arsenal of antiquity. Thus, for example, in his encyclopaedia and pharmacopoeia *De Materia Medica*, Pedanius Dioscorides (ca. 40–90 AD) mentions the ophthalmological use of cuttlefish bone to treat leukoma in livestock, applying the powder obtained from it to the eyes of sick animals –Book II, chapter 21– [116, 117]. In turn, in his encyclopaedic work *Naturalis Historia* Pliny the Elder (23–79 AD) also indicated that this powder was able to cure cataracts (Book XXXII, chapter 71), although he did not specify whether it was a useful therapy for both humans and animals [118, 119]. The *Hortus Sanitatis*, attributed to Johannes de Cuba [120], is a good work for understanding ethnoveterinary knowledge in antiquity, since it is the last encyclopaedic book on medical matters written in Latin and the first incunabulum dealing with these issues. Continuing with cuttlebone, in this work we hear from Aristotle that when mixed with salt it is a good remedy for curing white ulcers in the eyes of people and animals [121]. In a more agronomic context, one could mention, among others, Lucius Junius Moderatus Columella (4 – ca. 70 AD). In his work *De re rustica* we find remedies such as those prepared with honey that were applied to combat indigestion, lameness, pains and eye conditions [122].

In general it may be affirmed that the therapeutic indications described by Aristotle, Pliny and Dioscorides were used by renowned *albéitares* (the name formerly given to veterinarians and that is still used in some parts of Spain, especially in rural settings), such as Francisco de la Reina and Fernando Calvo in the sixteenth century, Martín Arredondo in the seventeenth, or Fernando de Sande Lago in the eighteenth; and in the history of Spanish veterinary medicine it should be noted that during the reign of Isabel II (1833–1868), among the extra-official therapeutic resources the most widely used ones were honey and leeches. Moreover, for this purpose the Royal Apothecary dispensed products based on invertebrates, in particular medicinal oils such as scorpion oil, blister beetle oil or earthworm oil [123]. Undoubtedly, the parallelism between human and veterinary medicine in medical systems in both the official and popular contexts can be seen in Spain throughout its history [22, 35, 124, 125].

In comparison with ethnoveterinary data gathered recently by other authors for other Mediterranean countries [20, 126–129], it should first be noted that the 16 invertebrate species (and five ethnotaxa) used in contemporary Spanish EVM constitute a very high number of zootherapeutic resources. Another relevant aspect is that we only observed coincidence in the use of cobwebs as an embrocation for skin injuries in horses and cuttlefish bone against eye infections in sheep. Bartha *et al*. [129] have documented the use of cobweb for wounds in Transylvania. For their part, Piluzza *et al*. [20] have recorded the use by shepherds in certain rural areas of Sardinia of powdered cuttlefish bone, which they blow into the eyes of the animals or massage it into them. This medicinal product of animal origin has been used in traditional healing since antiquity. For example, Lev [1] mentions cuttlefish bone as a remedy for human skin, eye and tooth diseases in the early Muslim and Crusader periods and in the late Ottoman period; this remedy is still in use in the twenty-first century.

By contrast, with respect to Latin America we also find a greater parallelism in the invertebrate groups and body parts used and the animal ailments treated. Honey from *Apis mellifera* is used in Paraíba State (NE Brazil) to treat eye problems in domestic animals in general, especially blindness and inflammations, and colds in cattle [33]. The *óleo-de-bicho*, the oil obtained by frying the larvae of the weevil *Rhynchophorus palmarum* (Linnaeus, 1758), has also been reported as being a medicinal agent in the EVM of the Marajó Island (Eastern Amazonia, Brazil). The main applications of this medicinal oil are wound healing and anti-inflammatory treatments [130].

## Conclusions

This review concerning the ethnoveterinary use of invertebrates reveals that humans have always considered this animal group as a source of surprising and numerous therapeutic properties, and it shows that a plethora of invertebrate-based remedies has been amassed in Spanish EVM. A high diversity of animal parts and derivative products are used and this is a heritage that could constitute a fundamental step for the discovery and isolation of natural extracts from animals in the search for new and low-cost drugs for livestock, in particular alternative drugs to others that elicit undesired side effects or are subject to a progressive loss of efficacy owing to the development of resistance. Likewise, the data documented also invite further research to determine the validity of these folk remedies.

In Spain the literature on EVM is incipient and the need for new studies is clear, mainly considering the cultural, socio-economic and ecological importance associated with the zoological resources used. Owing to the progressive loss of local veterinary knowledge this is also an urgent matter. The uses, practices and rituals documented only survive in the memories of elderly people, such that it would be highly recommendable to hold workshops or meetings with elderly people from the rural setting to conserve ethnozoological heritage.

Knowledge of the species catalogued may have other cultural and scientific applications, such as the development of educational or biodiversity conservation and management programmes [131], and they could also offer a solid grounding for future ethnozoological studies.

The need to conserve and protect medicinal invertebrates, animals that are rarely included in the national or international lists of threatened species, is required not only for humans but also for their domesticated animals.
